# Supplementary material for: Causal Relationship between Aging and Anorexia Nervosa: A White-Matter-Microstructure-Mediated Mendelian Randomization Analysis
Source: Biomedicines. 2024 Aug 16;12(8):1874. doi: 10.3390/biomedicines12081874 (PMC11351342; doi:10.3390/biomedicines12081874)
Supplement: Supplementary file 1 [file biomedicines-12-01874-s001.zip › Supplementary Figure/Figure S4/Singe SNP plot of MR analysis for TL to ubm-b-1843.pdf]

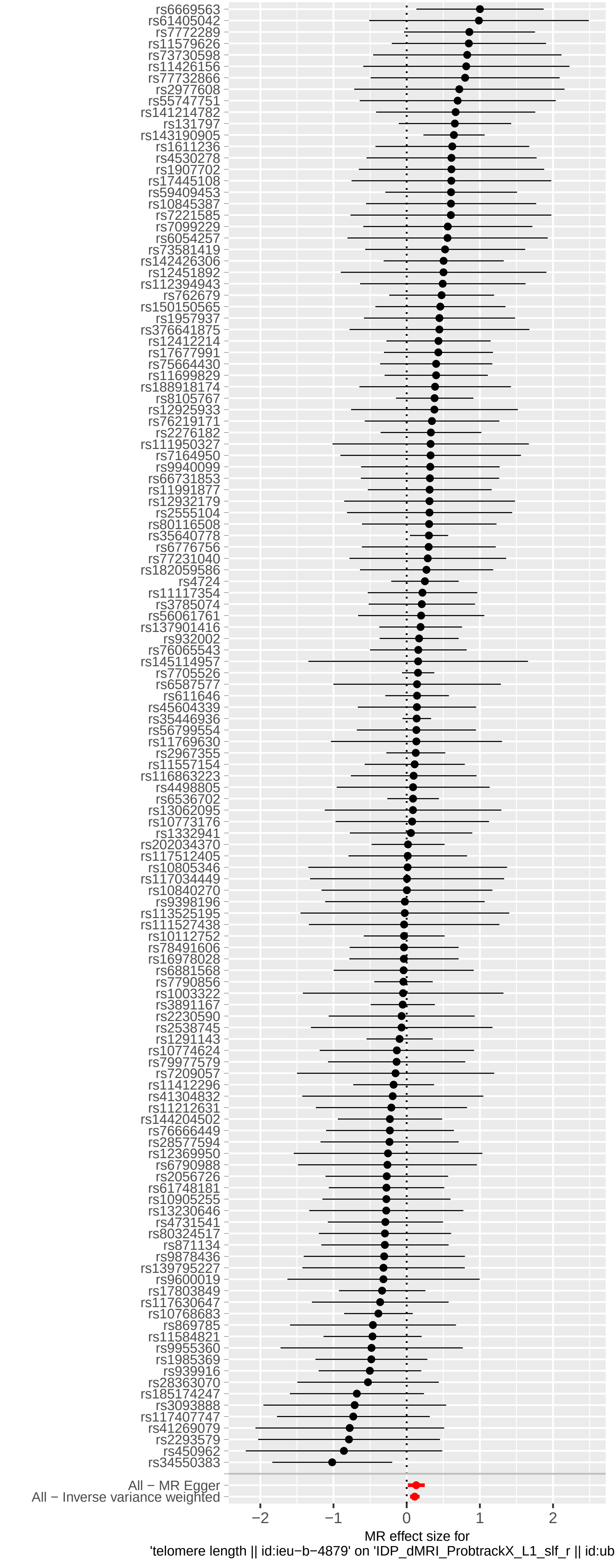
rs6669563
rs61405042
rs7772289
rs11579626
rs73730598
rs11426156
rs77732866
rs2977608
rs55747751
rs141214782
rs131797
rs143190905
rs1611236
rs4530278
rs1907702
rs17445108
rs59409453
rs10845387
rs7221585
rs7099229
rs6054257
rs73581419
rs142426306
rs12451892
rs112394943
rs762679
rs150150565
rs1957937
rs376641875
rs12412214
rs17677991
rs75664430
rs11699829
rs188918174
rs8105767
rs12925933
rs76219171
rs2276182
rs111950327
rs7164950
rs9940099
rs66731853
rs11991877
rs12932179
rs2555104
rs80116508
rs35640778
rs6776756
rs77231040
rs182059586
rs4724
rs11117354
rs3785074
rs56061761
rs137901416
rs932002
rs76065543
rs145114957
rs7705526
rs6587577
rs611646
rs45604339
rs35446936
rs56799554
rs11769630
rs2967355
rs11557154
rs116863223
rs4498805
rs6536702
rs13062095
rs10773176
rs1332941
rs202034370
rs117512405
rs10805346
rs117034449
rs10840270
rs9398196
rs113525195
rs111527438
rs10112752
rs78491606
rs16978028
rs6881568
rs7790856
rs1003322
rs3891167
rs2230590
rs2538745
rs1291143
rs10774624
rs79977579
rs7209057
rs11412296
rs41304832
rs11212631
rs144204502
rs76666449
rs28577594
rs12369950
rs6790988
rs2056726
rs61748181
rs10905255
rs13230646
rs4731541
rs80324517
rs871134
rs9878436
rs139795227
rs9600019
rs17803849
rs117630647
rs10768683
rs869785
rs11584821
rs9955360
rs1985369
rs939916
rs28363070
rs185174247
rs3093888
rs117407747
rs41269079
rs2293579
rs450962
rs34550383
All − MR Egger
All − Inverse variance weighted
−2
−1
0
1
2
MR effect size for
'telomere length || id:ieu−b−4879' on 'IDP_dMRI_ProbtrackX_L1_slf_r || id:ub
